# HIF1α-Dependent Metabolic Signals Control the Differentiation of Follicular Helper T Cells

**DOI:** 10.3390/cells8111450

**Published:** 2019-11-17

**Authors:** Lin Dong, Ying He, Shuping Zhou, Yejin Cao, Yan Li, Yujing Bi, Guangwei Liu

**Affiliations:** 1 Key Laboratory of Cell Proliferation and Regulation Biology, Ministry of Education, Institute of Cell Biology, College of Life Sciences, Beijing Normal University, Beijing 100875, China; 201731200020@mail.bnu.edu.cn (L.D.); 201821200008@mail.bnu.edu.cn (Y.H.); 201921200005@mail.bnu.edu.cn (Y.C.); liyan1106369@163.com (Y.L.); 2 Institute of Basic Medicine, Shandong First Medical University & Shandong Academy of Medical School, Jinan 250062, China; shupzhou718@163.com; 3 State Key Laboratory of Pathogen and Biosecurity, Beijing Institute of Microbiology and Epidemiology, Beijing 100071, China

**Keywords:** follicular helper T cell, T cell differentiation, HIF1α, glycolysis, oxidative phosphorylation, virus infection, infectious inflammation, GC responses, B cell immunity

## Abstract

Follicular helper T (T_FH_) cells are critical for germinal center (GC) formation and are responsible for effective B cell-mediated immunity; metabolic signaling is an important regulatory mechanism for the differentiation of T_FH_ cells. However, the precise roles of hypoxia inducible factor (HIF) 1α-dependent glycolysis and oxidative phosphorylation (OXPHOS) metabolic signaling remain unclear in T_FH_ cell differentiation. Herein, we investigated the effects of glycolysis and OXPHOS on T_FH_ cell differentiation and GC responses using a pharmacological approach in mice under a steady immune status or an activated immune status, which can be caused by foreign antigen stimulation and viral infection. GC and T_FH_ cell responses are related to signals from glycolytic metabolism in mice of different ages. Foreign, specific antigen-induced GC, and T_FH_ cell responses and metabolic signals are essential upon PR8 infection. Glycolysis and succinate-mediated OXPHOS are required for the GC response and T_FH_ cell differentiation. Furthermore, HIF1α is responsible for glycolysis- and OXPHOS-induced alterations in the GC response and T_FH_ cell differentiation under steady or activated conditions *in vivo*. Blocking glycolysis and upregulating OXPHOS signaling significantly recovered T_FH_ cell differentiation upon PR8 infection and ameliorated inflammatory damage in mice. Thus, our data provide a comprehensive experimental basis for fully understanding the precise roles of HIF1α-mediated glycolysis and OXPHOS metabolic signaling in regulating the GC response and T_FH_ cell differentiation during stable physiological conditions or an antiviral immune response.

## 1. Introduction

Follicular helper T (T_FH_) cells are specialized T helper (T_H_) cells that localize in the follicles and germinal center (GC) and can selectively stimulate B cells for GC responses, contributing to immunoglobulin production and memory B cell and long-lived plasma cell development [1,2]. T_FH_ cells express some migration-related molecules, including the chemokine receptor CXCR5, the inducible costimulatory molecule ICOS, the T cell inhibitory receptor PD-1, and the cytokine IL-21 [3,4]. GCs are transient and essential structures in B cell follicles of secondary lymphoid tissue [5,6,7]. T_FH_ cells control GC formation and maintenance, which should help B cell maturation, positive selection and differentiation into memory B cells, and should help long-lived B cells to efficiently mediate humoral immunity [8,9]. Bcl-6 is a master-specific transcriptional factor for T_FH_ cells [10,11,12]. IL-6 and IL-21 contribute to the differentiation of T_FH_ cells. ICOS and its ligand are required for Bcl-6 expression and T_FH_ cell differentiation [13,14,15]. In contrast, IL-2 potently inhibited T_FH_ cell differentiation through the transcription factors STAT5 and Blimp1, which can significantly inhibit the expression of Bcl-6 [16,17,18].

T cell differentiation is accompanied by dynamic metabolic reprogramming [19]. It has been shown that initial T cell activation and differentiation require naïve T cells to reprogram their metabolic status by shutting down oxidative phosphorylation (OXPHOS) and engaging in different metabolic pathways, including glycolysis and the pentose phosphate pathway, to meet the bioenergy demands [20,21]. Once cells enter the terminal differentiation, memory T cells instead use OXPHOS to provide the energy needed by the body [21,22,23,24]. These energy metabolism modes have obviously remodeled mitochondrial structure and respiratory capacity, which can obviously meet larger energy needs [24]. Energy metabolism patterns are different at different developmental and activation stages of T cells, and different T cell subsets often have distinct energy metabolism patterns. Although studies have shown that T_FH_ cell-mediated humoral immunity is often mediated by glucose metabolism signals, the precise roles of hypoxia inducible factor (HIF) 1α-mediated glycolysis and OXPHOS metabolic signals remain unclear.

In this study, we used pharmacological methods to observe the effects of glycolysis and OXPHOS on GC responses and T_FH_ cell differentiation under steady status or activated status, such as foreign antigen stimulation and viral infection. It was found that HIF1α-dependent glycolysis and OXPHOS are important for GC responses and T_FH_ cell differentiation and play critical roles in anti-virus infectious immunity.

## 2. Materials and Methods

### 2.1. Mice and Treatments

The wild-type (WT) B6 mice used in the experiments were mostly 8 weeks old unless otherwise indicated in the figure legend and were obtained from Beijing Weitonglihua Experimental Animal Center. *Hif1α*^flox/flox^ mice (on the B6 genetic background) were crossed with *Cd4-cre* mice to obtain *Hif1α*^-/-^ mice, as described previously [25,26]. All animal experiments were performed in accordance with protocols (CLS-EAW-2016-014) approved by the Animal Ethics Committee of Beijing Normal University on May 30, 2016. WT mice were treated as previously described [27]. Briefly, WT mice were injected intraperitoneally (i.p.) with diethyl succinate ( succinate, Suc, Sigma-Aldrich, St. Louis, USA; 70 mg/kg/mouse) or 2-deoxy-D-glucose (2-DG, Santa Cruz Biotechnology, Dallas, Texas, USA; 200 mg/kg/mouse) diluted in phosphate-buffered saline (PBS) in a volume of 200 μL daily for 8 consecutive days.

### 2.2. Mice Immunized with Ovalbumin (OVA)

WT or *Hif1α*^-/-^ mice were immunized by i.p. injection with OVA (Sigma-Aldrich; 100 μg) plus lipopolysaccharide (LPS, Sigma-Aldrich; 10 μg) in alum (Thermo Fisher Scientific, MS, Madison WI, USA; 200 μL each mouse), as described previously [28]. After 8 days, we determined the GC responses and T_FH_ cell differentiation in the mouse spleens.

### 2.3. Influenza Virus PR8 Infection

WT mice were infected intranasally with the mouse-adapted influenza virus (PR8, H1N1) at a dose of 450 TCID50 (the half maximal tissue culture infectious dose) in a volume of 50 μL (each mouse), as described previously [29]. After 8 days, we analyzed the GC response and T_FH_ cell differentiation in bronchoalveolar lavage fluid (BALF) and lungs of infected mice.

### 2.4. Histology

Lungs were fixed in 4% paraformaldehyde and embedded in paraffin, sectioned at 4 μm, mounted on positively charged glass slides (Superfrost Plus), and dried at 60 °C for 20 min. Hematoxylin and eosin (H&E) staining was completed by Wuhan Servicebio Co., Ltd., in China. Photographic analysis of H&E sections was performed with a ZEISS positive fluorescence microscope (Imager M1 microscopy, Carl Zeiss, Oberkochen, Baden-Württemberg, Germany), as described previously [30].

### 2.5. Flow Cytometry

The cell surface markers and cytokines were analyzed by flow cytometry, as described previously [30,31,32]. Living cells were stained with the following antibodies in PBS containing 0.1% (weight/volume) bovine serum albumin and 0.1% NaN_3_ for 30 min on ice. The following antibodies were obtained from BD Biosciences (Lake Franklin, NJ, USA): anti-CD4 (GK1.5), anti-B220 (RA36B2), anti-CXCR5 (2G8), anti-T- and B-cell activation antigen (GL-7), anti-CD95 (Jo2), anti-CD138 (281-2), and anti-IgD (11-26c.2a). The following antibodies were obtained from eBioscience (Thermo Fisher): anti-PD-1 (J43), anti-CXCR5 (SPRCL5), and anti-IL-21 (FFA21). To detect cytokine secretion, cells were stimulated with phorbol-12-myristate-13-acetate (PMA; Sigma-Aldrich) and ionomycin (PeproTech-BioGems, NJ, USA) for 5 hours. The cells were fixed using a Fixation/Permeabilization Solution Kit (BD Biosciences). To detect the metabolism-related regulators, we used anti-Glut1 (EPR3915) and anti-SDHα (EPR9043B) antibodies. Cells were fixed with a Fixation/Permeabilization Solution Kit (BD Biosciences) and were intracellularly stained for both molecules. All flow cytometry data were obtained with ACEA NovoCyte (ACEA Biosciences, Inc., San Diego, CA, USA), and the data were analyzed with NovoExpress (TreeStar, San Carlos, CA).

### 2.6. Metabolic Assays

The respiratory burst indicated by the proton production rate (PPR) and the oxygen consumption rate (OCR) were measured as previously described [32,33]. Briefly, T_FH_ cells were sorted from the spleen. PPR and OCR were measured with an XF_e_24 extracellular flux analyzer (Seahorse Bioscience, Agilent Technologies, Inc, Palo Alto, CA, USA) according to the manufacturer’s instructions as described previously [25,34]. In brief, T_FH_ cells were sorted from splenocytes and seeded into XF_e_24 microplates (2 × 10^5^) to immobilize the cells. The cells were washed with XF base medium (assay medium, Sigma-Aldrich) with glucose (10 mM), sodium pyruvate (1 mM), and L-glutamine (2 mM). After incubation in the assay medium in an incubator without CO_2_ for 1 hour, cells were subjected to oxygen consumption assays with a Mito Stress Test Kit (Seahorse Biosciences). Oligomycin (Sigma-Aldrich; 1 μM), FCCP (mitochondrial oxidative phosphorylation uncoupler, Sigma-Aldrich; 1 μM), and rotenone/antimycin A (Sigma Aldrich; 0.5 μM) were added to the medium in order. The data were acquired on Seahorse XF-24 and analyzed using Wave.

### 2.7. Quantitative Real-Time PCR

RNA was extracted with TRIzol reagent (Sigma-Aldrich) in T_FH_ cells (CXCR5^+^PD1^+^CD4^+^T cells) sorted from the spleen, BALF, or lung. Complementary DNA (cDNA) was synthesized using the PrimeScript™ RT Master Mix (Perfect Real Time; TaKaRa, Osaka City, Osaka Prefecture, Japan). An ABI Q6 Flex Real-time PCR system (ThermoFisher Scientific) was used for quantitative PCR with primers from Applied Biosystems (Carlsbad, CA, USA). The *Glut1* gene-specific primers used in this study are as follows: forward primer, 5′-cagctgtcgggtatcaatgc-3′; reverse primer, 5′-tccagctcgctctacaacaa-3′. The *Sdha* gene-specific primers used in this study are as follows: forward primer, 5′-tgctgggtacttgaatccct-3′; reverse primer, 5′-atgaacgtagtcggtaaccac-3′. The individual gene expression was calculated and normalized to the expression of *Hprt*. The primers used for *Hprt* were as follows: forward primer, 5′-agtacagccccaaaatggttaag-3′; reverse primer, 5′-cttaggctttgtatttggcttttc-3′. To determine the relative quantities, SYBR^®^ Premix ExTaq^TM^ (Perfect Real Time, TaKaRa) was used. The results were analyzed with an ABI Q6 Flex Real-time PCR system (ThermoFisher Scientific), as described previously [26].

### 2.8. Statistical Analyses

All data are presented as the means ± SDs. Student’s unpaired *t* test was used to compare two sets of parametric data. When comparing three or more datasets, one-way analysis of variance with Dunnett’s post hoc test was applied for parametric data, and a Kruskal-Wallis test was applied for nonparametric data; *p* < 0.05 was considered to be statistically significant.

## 3. Results

### 3.1. GC and T_FH_ Cell Responses in Mice of Different Ages Are Related to Signals from Glycolytic Metabolism

**Figure 1:**
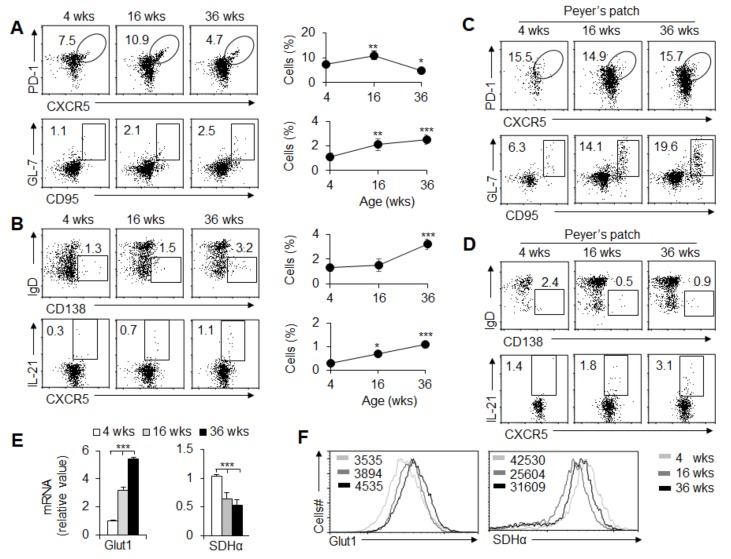
Age-related GC responses and T_FH_ cell differentiation. (**A**) Flow cytometry of T_FH_ cells (CXCR5^+^PD-1^+^) among CD4^+^ T cells and GC B cells (CD95^+^GL-7^+^) among B220^+^ cells in spleens from wild-type (WT) mice at the ages of 4, 16, and 36 weeks. The right panel shows the frequency of T_FH_ cells and GC B cells. (**B**) Flow cytometry of plasma cells (IgD^-^CD138^+^) among B220^+^ cells and IL-21^+^ T_FH_ cells in spleens. The right panel shows the frequency of plasma cells and IL-21^+^T_FH_ cells. (**C**) Flow cytometry of T_FH_ cells and GC B cells in Peyer’s patches (PPs) from WT mice at 4, 16, and 36 weeks of age. (**D**) Flow cytometry of plasma cells and IL-21^+^T_FH_ cells in PPs. (**E**) *Glut1* and *Sdhα* mRNA expression was examined by real-time PCR analysis in T_FH_ cells sorted from the splenocytes. (**F**) Flow cytometry of Glut1 and SDHα expression in T_FH_ cells in spleens. Analyses of mean fluorescence intensity (MFI) are shown. Data are representative of three individual experiments (n = 3–6 mice per group). * *p* < 0.05; ** *p* < 0.01; *** *p* < 0.001, compared with the indicated groups.

We first explored the GC and T_FH_ cell response in peripheral immune organs in mice of different ages (weeks). Under a steady state, the spleens were obtained from mice of different ages (4, 16, and 36 weeks old). Spleens from 4-week-old mice contained a population of T cells expressing the T_FH_ cell markers PD-1 and CXCR5 and B cells expressing the GC markers GL-7 and CD95; the T_FH_ cells and GC B cell frequencies were markedly enhanced with age from 4 weeks old to 16 weeks old. After that, T_FH_ cells decreased significantly, while GC B cells continued to increase in the 36-week-old mice (Figure 1A). Furthermore, IgD^-^CD138^+^ plasma B cells were significantly increased in mice from 4 weeks old to 36 weeks old (Figure 1B). IL-21 is critical for T_FH_ cell differentiation and function, and we found that IL-21 production in T_FH_ cells also showed a consistent tendency with age (Figure 1B). Therefore, GC and T_FH_ responses have age-related characteristics, but T_FH_ and GC reactions show different tendencies in peripheral immune tissue.

Generally, the peripheral immune organs are stimulated by foreign antigens to induce a GC response, but this is very special in Peyer’s patches (PPs). In PPs, GC responses are continuously present, which is very important for the secretion of intestinal immunoglobulin to maintain the intestinal immune homeostasis. The spontaneous GC responses are maintained by long-term exposure to intestinal microorganisms and strictly depend upon the assistance of T_FH_ cells [35,36]. PPs in mice that ranged from 4 weeks old to 36 weeks old showed enhanced frequencies of T_FH_ cells and GC B cells (Figure 1C), and the IL-21 secretion in T_FH_ cells was markedly enhanced with age (Figure 1D). However, the IgD^-^CD138^+^ plasma B cells were markedly decreased with age (Figure 1D), which indicates that the intestinal mucosal B cell response probably shows different characteristics from peripheral immune organs in mice.

The metabolic requirements of T_FH_ cell differentiation are not well understood. To investigate the precise roles of glycolysis and the OXPHOS metabolic pathway in T_FH_ cell differentiation, we determined Glut1, a key regulator of glycolysis. Additionally, succinate dehydrogenase (SDH) supports metabolic repurposing of T cell differentiation and functional activity [1]. We also determined the level of SDHα, a key enzyme subset of SDH during the OXPHOS metabolic pathway. *Glut1* mRNA and protein levels in T_FH_ cells were markedly enhanced with an age-dependent tendency, but *Sdhα* mRNA and protein levels were markedly downregulated with an age-dependent tendency (Figure 1E,F). These data collectively showed that T_FH_ cell differentiation was probably related to glycolysis and the OXPHOS metabolic signal in mice of different ages.

### 3.2. Foreign Antigen-Induced GC and T_FH_ Cell Responses in an Antigen-Specific Manner

**Figure 2:**
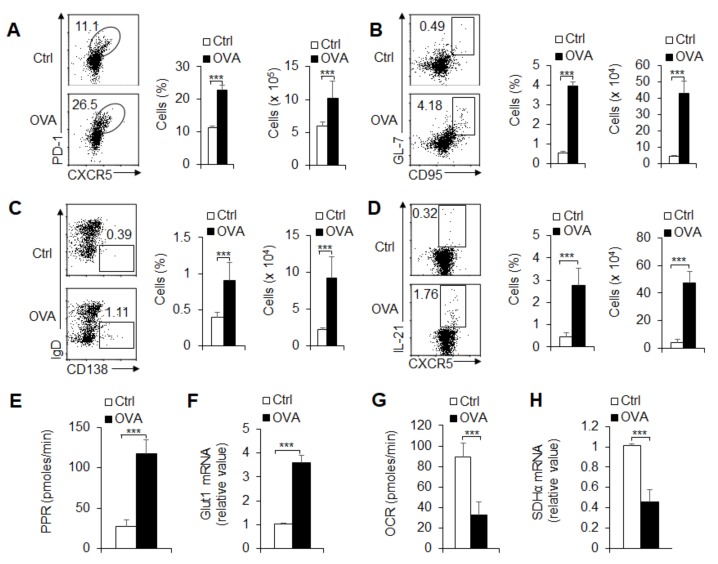
T_FH_ cell differentiation is associated with glycolysis and OXPHOS with OVA immunization. WT mice underwent intraperitoneal immunization with OVA plus LPS in alum at 8 days, and spleens were isolated for analysis. (**A**) Flow cytometry of T_FH_ cells in spleens. The right panel shows the frequency and the number of T_FH_ cells. (**B**,**C**) Flow cytometry of GC B cells and plasma cells in spleens; the statistical results are also shown. (**D**) The secretion of IL-21 in CXCR5^+^PD1^+^CD4^+^T_FH_ cells in spleens. The right panel shows the frequency and the number of IL-21^+^ T_FH_ cells. (**E**) The proton production rate (PPR) was examined as a readout for glycolysis of CXCR5^+^PD1^+^CD4^+^T_FH_ cells sorted from splenocytes. (**F**) *Glut1* mRNA expression was analyzed by real-time PCR in CXCR5^+^PD1^+^CD4^+^T_FH_ cells sorted from splenocytes. (**G**) The oxygen consumption rate (OCR) was examined as a readout for OXPHOS of CXCR5^+^PD1^+^CD4^+^T_FH_ cells sorted from splenocytes. (**H**) *Sdhα* mRNA expression was analyzed by real-time PCR in CXCR5^+^PD1^+^CD4^+^T_FH_ cells sorted from the splenocytes. Data are representative of four individual experiments (n = 3–7 mice per group). *** *p* < 0.001, compared with the indicated groups.

Although T_FH_ and GC B cells showed different responses after age 16 weeks in peripheral immune organs, they showed a constant increase before age 16 weeks. Therefore, in the follow-up experiment, we selected 8-week-old mice to observe the response of GC B cells and T_FH_ cells. To investigate the GC and T_FH_ cell response under a cell-activated state, we immunized mice with OVA. Immunized mice had enhanced T_FH_ cell and GC cell frequency and number in the spleens (Figure 2A,B) and draining lymph nodes (data not shown). IgD^-^CD138^+^ plasma B cells and IL-21 production were markedly enhanced after immunization with OVA (Figure 2C,D).

T cell differentiation is accompanied by dynamic metabolic reprogramming. To evaluate the role of glycolysis and OXPHOS, we isolated T_FH_ cells from the spleen of immunized mice with OVA and examined the metabolic activity of the T_FH_ cells. The results showed that immunization leads to an increase in PPR and a reduction in OCR in activated T_FH_ cells, and these results suggest that antigen-specific immunization significantly elevates the rate of glycolysis but not OXPHOS (Figure 2E,G). Consistently, the key regulator Glut1 and SDHα of glycolysis and the OXPHOS metabolic pathway were markedly upregulated or downregulated in T_FH_ cells, respectively (Figure 2F,H). Thus, glycolysis and OXPHOS metabolic signals are related to GC responses and T_FH_ cell differentiation via antigen-specific mechanisms.

### 3.3. T_FH_ Cell Differentiation and Metabolic Signals Are Essential upon PR8 Infection

**Figure 3:**
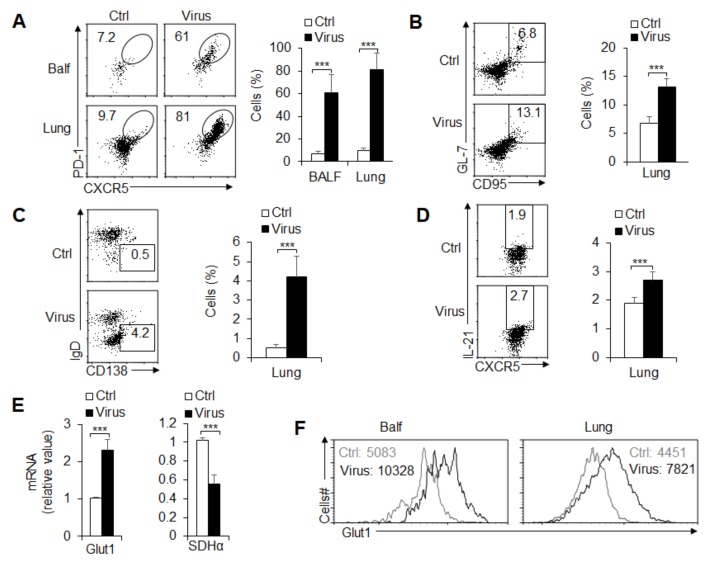
T_FH_ cell differentiation is associated with glycolysis and OXPHOS upon influenza virus PR8 infection. Mice infected with influenza H1N1 virus PR8-infected mice at 8 days and BALF and lungs were isolated for analysis. (**A**) Flow cytometry of T_FH_ cells in BALF and lungs. The right panel shows the frequency of T_FH_ cells. (**B**,**C**) Flow cytometry of GC B cells (B) and plasma cells (C) in lungs. The right panel shows the frequency of GC B cells and plasma cells. (**D**) The percent of IL-21^+^ T_FH_ cells in the lungs. The right panel shows the statistical results. (**E**) *Glut1* and *Sdhα* mRNA expression was analyzed by real-time PCR in CXCR5^+^PD1^+^CD4^+^T_FH_ cells sorted from BALF. (**F**) Flow cytometry analysis of the MFI of Glut1 expression in T_FH_ cells in BALF and lungs. Data are representative of three individual experiments (n = 3–6 mice per group). *** *p* < 0.001, compared with the indicated groups.

T_FH_ cell enrichment plays an important role in the regulation of antiviral infection [1,3]. Therefore, to emulate the GC reaction in response to acute viral infection, we challenged mice with the PR8 H1N1 virus strain. On day 8 after infection, the mice had enhanced T_FH_ cell and GC cell frequency and number in BALF and lung tissue (Figure 3A,B) and draining lymph nodes (data not shown). IgD^-^CD138^+^ plasma B cells and IL-21 production were markedly enhanced after PR8 infection (Figure 3C,D). Furthermore, the results showed that PR8 infection leads to an increase in PPR and a reduction in OCR in activated T_FH_ cells, which suggests that PR8 infection significantly elevates the rate of glycolysis but not that of OXPHOS (data not shown). Consistently, the key regulator Glut1 of glycolysis or key enzyme SDHα of the OXPHOS metabolic pathway was markedly upregulated or downregulated, respectively (Figure 3E,F; data not shown). Thus, glycolysis and OXPHOS metabolic signals are related to GC responses and T_FH_ cell differentiation upon viral infection.

### 3.4. Glycolysis Is Required for GC Response and T_FH_ Cell Differentiation

**Figure 4:**
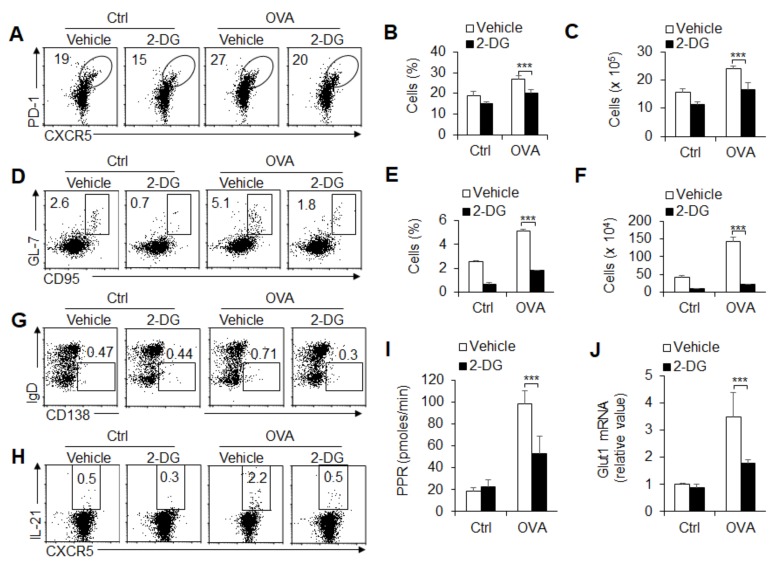
Blocking glycolysis inhibits T_FH_ cell differentiation stimulated by foreign antigens. WT mice underwent intraperitoneal immunization with OVA plus LPS in alum and in the presence of PBS (vehicle) or 2-DG (200 mg/kg/day) i.p. at 8 days, and mouse spleens were isolated for analysis. (**A**–**C**) Flow cytometry of T_FH_ cells in spleens in A. The frequency and the number of T_FH_ cells are shown in B–C. (**D**–**F**) Flow cytometry of GC B cells in spleens in D. The frequency and the number of GC B cell are shown in E and F. (**G**,**H**) Flow cytometry analysis of plasma cells (**G**) and IL-21^+^T_FH_ cells (H) in spleens. (**I**) PPR was analyzed as a readout for glycolysis in T_FH_ cells sorted from splenocytes. (**J**) *Glut1* mRNA expression was analyzed by real-time PCR in T_FH_ cells sorted from splenocytes. Data are representative of four individual experiments (n = 3–5 mice per group). *** *p* < 0.001, compared with the indicated groups.

To further investigate the role of glycolysis in GC response and T_FH_ cell differentiation, these mice were immunized with OVA and treated with glycolysis inhibitor 2-DG, 2-DG alone or OVA immunization alone *in vivo*. The results showed that OVA immunization markedly enhanced T_FH_ cell differentiation and GC responses, but 2-DG combined with OVA immunization treatment significantly recovered these alterations (Figure 4A–F). Furthermore, IgD^-^CD138^+^ plasma B cells and IL-21 production were markedly enhanced after immunization with OVA, but 2-DG combined with OVA immunization treatment significantly recovered these alterations (Figure 4G,H and Supplementary Figure S1). Moreover, 2-DG treatment significantly blocked PPR levels and *Glut1* expression (Figure 4I,J), which indicates that 2-DG treatment efficiently blocked the glycolysis signaling pathway when these mice were immunized with a foreign antigen. Together, these results suggest that glycolysis signaling is critically required for GC response and T_FH_ cell differentiation under foreign antigen stimulation.

### 3.5. Succinate-Mediated OXPHOS Is Sufficient for GC Response and T_FH_ Cell Differentiation

**Figure 5:**
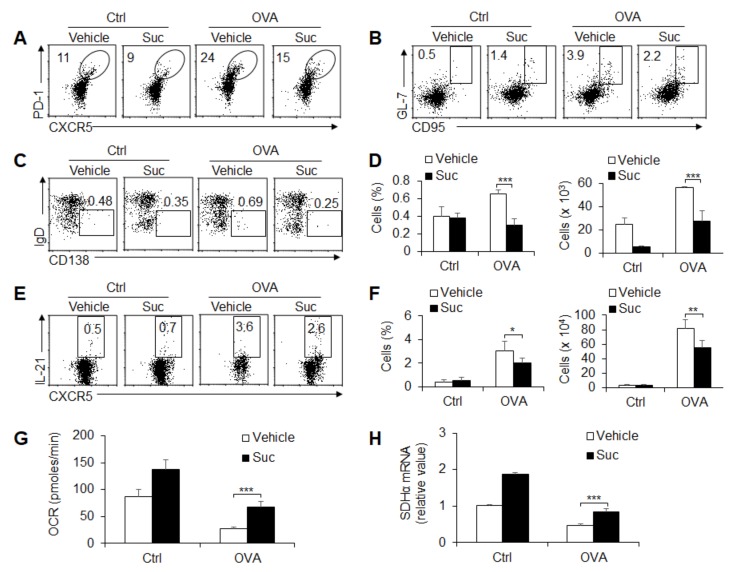
OXPHOS signaling controls T_FH_ cell differentiation induced by foreign antigens. WT mice underwent intraperitoneal immunization with OVA plus LPS in alum and in the presence of PBS (vehicle) or succinate (Suc, 70 mg/kg/day) i.p. at 8 days, and mouse spleens were isolated for analysis. (**A**) Flow cytometry of T_FH_ cells in spleens. (**B**) Flow cytometry of GC B cells in spleens. (**C**,**D**) Flow cytometry analysis of plasma cells (**C**) and the frequency and number of plasma cells are shown (**D**). (**E**,**F**) Flow cytometry analysis of IL-21^+^CXCR5^+^PD1^+^CD4^+^T_FH_ cells in spleens (**E**). The frequency and the number of IL-21^+^ T_FH_ cells are shown (**F**). (**G**) OCR was analyzed as a readout for OXPHOS in T_FH_ cells sorted from splenocytes. (**H**) *Sdhα* mRNA expression was analyzed by real-time PCR in CD4^+^T_FH_ cells sorted from splenocytes. Data are representative of three individual experiments (n = 4–5 mice per group). *** *p* < 0.001, compared with the indicated groups.

SDH and SDH-mediated OXPHOS are critical for T cell differentiation and metabolic reprogramming in immunity [1,2]. To further investigate the role of succinate and OXPHOS signaling in the GC response and T_FH_ cell differentiation, these mice were immunized with OVA and treated with SDH activator diethyl succinate (Suc) or Suc alone or OVA immunization alone *in vivo*. The results showed that OVA immunization markedly enhanced T_FH_ cell differentiation and GC responses, but Suc combined with OVA immunization treatment significantly recovered these alterations (Figure 5A,B). Furthermore, IgD^-^CD138^+^ plasma B cells and IL-21 production were markedly enhanced after immunization with OVA, but Suc combined with OVA immunization treatment significantly recovered these alterations (Figure 5C–F). Moreover, Suc combined with OVA immunization treatment significantly enhanced the OCR levels and *Sdhα* expression (Figure 5G,H), which indicates that Suc treatment efficiently enhanced the SDH levels and OXPHOS signaling activities when these mice were immunized with a foreign antigen. Together, these results suggest that succinate and OXPHOS signaling are sufficient for the GC response and T_FH_ cell differentiation under foreign antigen stimulation.

### 3.6. HIF1α-Dependent Glycolysis and OXPHOS Are Required for GC Response and T_FH_ Cell Dfferentiation

**Figure 6:**
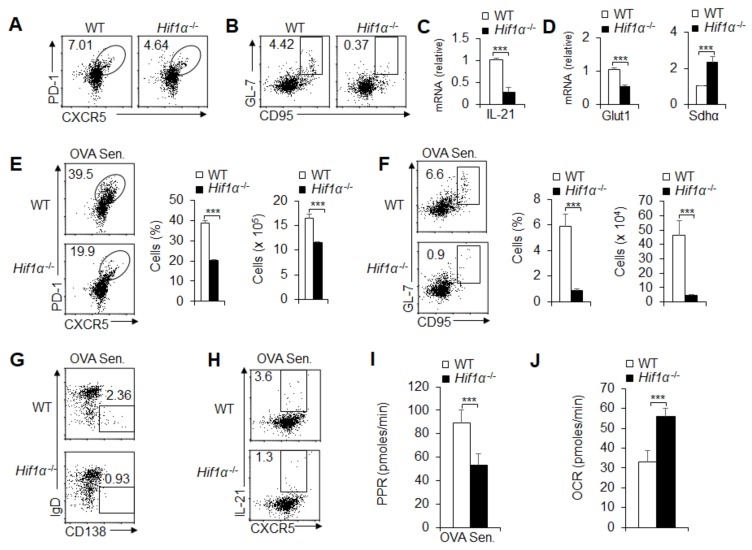
HIF1α is responsible for glycolysis and OXPHOS in T_FH_ cell differentiation and GC responses. (**A**,**B**) Flow cytometry of T_FH_ cells (**A**) and GC B cells (**B**) in spleens from WT and *Hif1α*^-/-^ mice. (**C**) *Il21* mRNA expressions of T_FH_ cells sorted from splenocytes in WT and *Hif1α*^-/-^ mice. (**D**) *Glut1* and *Sdhα* mRNA expressions of T_FH_ cells sorted from splenocytes in WT and *Hif1α*^-/-^ mice. (**E**–**J**) WT and *Hif1α*^-/-^ mice underwent intraperitoneal OVA immunization (OVA Sen.) with OVA plus LPS in alum at 8 days, and mouse spleens were isolated for analysis. (**E**,**F**) Flow cytometry of T_FH_ cells and GC B cells in spleens from WT and *Hif1α*^-/-^ mice after immunization. The frequency and the number of T_FH_ cells and GC B cells are shown in the right panel. (**G**,**H**) Flow cytometry of plasma cells and IL-21^+^ T_FH_ cells in spleens from WT and *Hif1α*^-/-^ mice after immunization. (**I**–**J**) PPR (**I**) and OCR (**J**) were analyzed as the readout of glycolysis and OXPHOS in T_FH_ cells sorted from splenocytes in WT and *Hif1α*^-/-^ mice after immunization, respectively. Data are representative of four individual experiments (n = 4–5 mice per group). *** *p* < 0.001, compared with the indicated groups.

HIF1α is reported to be a key regulator of the activity of the glycolytic pathway in immune cells. Our previous studies also demonstrated that HIF1α is critically involved in macrophage polarization and metabolism [25,37]. Here, we further explored the role of HIF1α in T_FH_ cell differentiation and the glycolytic pathway activity. We crossed mice bearing loxp flanked alleles encoding *Hif1α* (*Hif1α*^fl/fl^) with *Cd4*-*Cre* mice to generate *Hif1α*^fl/fl^; *Cd4-Cre* mice (*Hif1α* gene specifically deleted in T cells). The results showed that under steady state, HIF1α deficiency markedly decreased the T_FH_ cell frequency and GC cell frequency (Figure 6A,B). Consistently, IL-21 production and *Glut1* mRNA expression were markedly lowered, and SDHα was markedly enhanced (Figure 6C,D). Furthermore, after stimulation with foreign antigens, although the value of T_FH_ cell frequency and GC cell frequency increased significantly, the frequency and number of T_FH_ cells and GC cells decreased significantly due to HIF1α deficiency (Figure 6E,F). Furthermore, IgD^-^CD138^+^ plasma B cells and IL-21 production were significantly lower in *Hif1*α^-/-^ mice compared with control WT compartments (Figure 6G,H). These data suggest that HIF1α is required for T_FH_ cell differentiation and GC responses under steady state or foreign antigen challenge in mice. Importantly, HIF1α deficiency markedly downregulated the PPR levels and upregulated the OCR levels in T_FH_ cells when challenged with foreign antigens in mice (Figure 6I,J and Supplementary Figure S2). Together, these data suggest that HIF1α-dependent glycolysis and OXPHOS are critically required for GC responses and T_FH_ cell differentiation under a steady and activated state in mice.

### 3.7. Alteration of Glycolysis and OXPHOS Signaling Controls T_FH_ Cell Differentiation upon PR8 Infection

**Figure 7:**
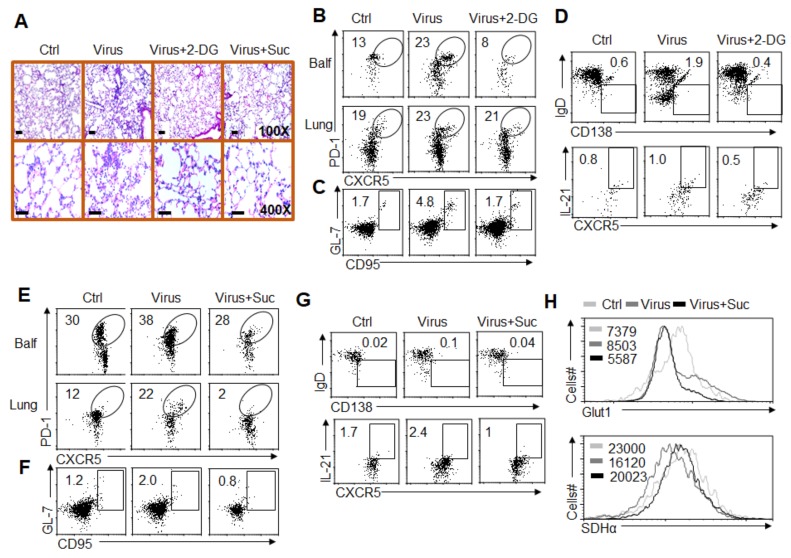
Alterations of glycolysis and OXPHOS signaling control T_FH_ cell differentiation upon PR8 infection. (**A**) Hematoxylin and eosin (H&E) histological staining of lung sections for general pathology and inflammatory cell infiltration at 8 days after PR8 infection and 2-DG (200 mg/kg/day) or succinate (Suc, 70 mg/kg/day) i.p. treatment. (**B**) Flow cytometry of T_FH_ cells in BALF and lung tissue from PR8-infected mice at 8 days in the presence of 2-DG treatment. (**C**,**D**) Flow cytometry of GC B cells (**C**), plasma cells and IL-21^+^ T_FH_ cells (**D**) in lungs from PR8-infected mice at 8 days in the presence of 2-DG treatment. (**E**) Flow cytometry of T_FH_ cells in BALF and lungs from WT mice at 8 days after PR8 infection and succinate treatment. (**F**,**G**) Flow cytometry of GC B cells (**F**), plasma cells and IL-21^+^ T_FH_ cells (**G**) in lungs from WT mice at 8 days after PR8 infection and succinate treatment. (**H**) Flow cytometry analysis of the MFI of Glut1 and SDHα expression in CXCR5^+^PD1^+^CD4^+^T_FH_ cells in lungs. Data are representative of four individual experiments (n = 4–7 mice per group).

T_FH_ cell recruitment and differentiation are critical for regulating the antiviral response *in vivo* [1,3]. Therefore, we further investigated the significance of HIF1α-dependent glycolysis and OXPHOS metabolic signaling in GC responses to T_FH_ cell differentiation upon PR8 infection. We pretreated WT mice with or without the glycolysis inhibitor 2-DG and challenged the mice with PR8 infections in vivo for 8 days. After 8 days of infection, the severity of infection was evaluated by measuring the pathological lung tissue damage with H&E staining. As shown in the figures, mice pretreated with 2-DG displayed a markedly ameliorated course of infection after the challenge (Figure 7 and Supplementary Figure S3). Microscopic and histological observations revealed markedly ameliorated pathological inflammation in lungs from mice pretreated with 2-DG (Figure 7A). Consistently, PR8 infection markedly enhanced the T_FH_ cell frequency and GC cell frequency in BALF and infected lung tissue. However, blocking glycolysis with 2-DG treatment significantly recovered these alterations (Figure 7B,C). Moreover, 2-DG treatments also significantly recovered the upregulated IL-21 production and IgD^-^CD138^+^ plasma B cell frequency challenged by virus infection (Figure 7D). Furthermore, blocking glycolysis with 2-DG did not alter the expression level of *Hif1α* mRNA during this course (Supplementary Figure S4), which suggests that HIF1α is an upstream molecule of the glycolysis signaling pathway. Together, these results suggest that glycolysis signaling is necessary for GC responses and T_FH_ cell differentiation upon PR8 infection.

We pretreated WT mice with or without the OXPHOS activator Suc and challenged the mice with a PR8 infection *in vivo* for 8 days. After 8 days of infection, the severity of infection was evaluated by measuring the pathological lung tissue damage with H&E staining. As shown in Figure 7A, mice pretreated with Suc displayed a markedly ameliorated course of infection after the challenge. Pathological observations revealed markedly ameliorated pathological inflammation in lungs from mice pretreated with Suc (Figure 7A). Consistently, PR8 infection markedly enhanced the T_FH_ cell frequency and GC cell frequency in infected lung tissue and BALF. However, upregulating OXPHOS signaling with Suc treatment significantly recovered these alterations (Figure 7,F). Moreover, Suc treatments also significantly recovered the upregulated IL-21 production and IgD^-^CD138^+^ plasma B cell frequency challenged by virus infection (Figure 7G,H). Together, these results suggest that succinate and OXPHOS signaling are essential for GC responses and T_FH_ cell differentiation upon PR8 infection.

## 4. Discussion

Metabolic reprogramming plays an important role in T cell activation, differentiation, and function [19,20,38]. On the one hand, cell metabolism provides enough energy for T cell development and differentiation; on the other hand, energy metabolites are also important regulators of the T cell response. Different T cell subsets often have different metabolic characteristics [16,39]. Therefore, targeting specific metabolic pathways can effectively treat immune-associated diseases mediated by different T cell subsets [40]. T_FH_ cells are a subset of CD4^+^T cells that specialize in helping B cells to produce antibodies in the face of antigenic challenge. T_FH_ cells have the unique ability to provide help to B cells for the formation of GC reactions and the development of humoral immunity [1]. Additionally, T_FH_ cells are essential for the generation of short- and long-lived humoral immunity, which is necessary for the protective response against a wide range of pathogens, including viruses [1,2]. Despite the recent identification of the metabolic regulation of T_FH_ cell differentiation, the precise mechanisms of glycolysis and OXPHOS in GC responses and T_FH_ cell differentiation under stable physiological status or pathological anti-virus immunity remain unknown. Herein, we showed that the GC and T_FH_ cell responses in mice of different ages are related to signals from glycolytic metabolism. Foreign antigen-induced, specific GC and T_FH_ cell responses, and metabolic signals are essential upon PR8 infection. Glycolysis and succinate-mediated OXPHOS are important for GC responses and T_FH_ cell differentiation. Additionally, HIF1α is responsible for glycolysis and OXPHOS alterations of the GC response and T_FH_ cell differentiation under steady or activated conditions *in vivo*. Blocking glycolysis and upregulating OXPHOS signaling significantly recovered T_FH_ cell differentiation upon PR8 H1N1 infection and ameliorated inflammatory damage in mice (Supplementary Figure S5). Altogether, our study illustrates that glycolysis and OXPHOS are differentially involved in mediating GC responses and T_FH_ cell differentiation under steady state and anti-virus immunity.

Glycolysis is the most universal pathway that converts glucose into pyruvate. However, OXPHOS occurs in the inner mitochondrial membrane of eukaryotic cells. It is a coupling reaction of the energy released during the oxidation of substances *in vivo* to supply ADP and inorganic phosphate to synthesize ATP through the respiratory chain [23,24]. Succinate oxidoreductase is the second enzyme of the electron transport chain (ETC). This is special because it is the only enzyme that belongs to both the tricarboxylic acid cycle (TCA) cycle and the ETC. Additionally, SDH-mediated OXPHOS supports metabolic repurposing of mitochondria to drive tumor cells or nontumor inflammatory macrophages and T cell differentiation and functional activity [41,42]. In the presence of oxygen, tumor cells also exhibit glycolysis, which is known as the Warburg effect [43]. In contrast, nontumor cells are often dependent on ambient oxygen concentrations and changes in cell metabolism from OXPHOS to glycolysis [44,45]. The activation or differentiation of naïve T cells is also closely related to metabolic reprogramming [19,46,47]. Naïve and memory T cells provide energy mainly through OXPHOS and fatty acid oxidation, which reflects their continuous low demand for energy [19]. In contrast, effector T cells require high-energy metabolism. These cells are similar to tumor cells in that they often provide energy through glycolysis [48]. Current research results have proven that different T cell subsets often require different metabolisms. Generally, T_H_1, T_H_2, T_H_9, and T_H_17 cells depend on glycolysis, while regulatory T cells (T_reg_) are more dependent on OXPHOS and fatty acid oxidation. Recent studies have shown that the T_FH_ cell response is associated with strong metabolic demands and have revealed some controversial roles of glycolysis during T_FH_ differentiation. T_FH_ secretes IL-21 by glycolysis *in vitro* [47], while Bcl-6 can inhibit T_FH_ cell activity by inhibiting gene expression in the glycolysis pathway [49,50]. Consistent with this, inhibition of the glycolysis pathway does not block Bcl6-expressing T_FH_ cell differentiation [51]. DUSP6 deficiency promoted T_FH_ cell differentiation and was associated with a severe defect in glycolysis [49]. mTOR positively controls T_FH_ cell differentiation and GC responses, which are associated with glycolysis and lipogenesis. Direct manipulation of metabolic activities through the transcription factors Myc and Glut1 modulated T_FH_ cell responses [52]. In this study, we evaluated the glycolytic activities of GC and T_FH_ cell responses and demonstrated that HIF1α-dependent glycolysis and succinate-mediated OXPHOS are positively and negatively associated with the ATP supply in the GC response and T_FH_ cell differentiation, respectively. In addition, we identified that glycolytic reprogramming plays a critical role in T_FH_ cell differentiation under antigen immunization or upon virus infection. 

HIF1α is a key regulator of the B cell adaptive immune response [53,54]. In the study of the human B-lymphocyte cell line under hypoxic conditions, HIF1α activated a series of glycolysis signal pathway gene transcription pathways, enhanced glycolysis activity, and produced ATP to meet the energy supply of B cells. Previously, we demonstrated that HIF1α-mediated activation of glycolysis had a beneficial effect for the synthesis of ATP under severely hypoxic conditions in directing T_H_9 cell differentiation in cancer during severe hypoxia or in noncancer T cells [26]. Here, we report that HIF1α-glycolysis and OXPHOS actively change the glycolytic activities of both OXPHOS and glycolysis, which play important roles in the GC and T_FH_ cell responses. These data suggest that glycolysis can take the form of active glycolysis without considering the activity of mitochondria, which is different from classical glycolysis during severe hypoxia. This active glycolysis may help to prevent excessive ROS production when mitochondrial respiration or OXPHOS is damaged. In addition, the dynamic balance between glycolysis and OXPHOS is helpful for reprogramming the GC and T_FH_ cell responses under physiological or pathological conditions.

In conclusion, our results showed that HIF1α-dependent glycolysis and OXPHOS processes significantly reprogrammed the GC responses and T_FH_ cell differentiation under steady state or antigen immunization conditions, even during an actual PR8 H1N1 infection. Therefore, these data collectively indicated that glycolysis and OXPHOS metabolic signaling are tightly linked to the GC response and T_FH_ cell differentiation. The precise metabolic profiles of GC responses and T_FH_ cell functional differentiation are intimately linked to their status and functions during antigen immunization and PR8 H1N1 infection.

## Supplementary Materials

The following are available online at https://www.mdpi.com/2073-4409/8/11/1450/s1, Supplementary Figure S1: Blocking glycolysis inhibits T_FH_ cell differentiation upon foreign antigen stimuli. Supplementary Figure S2: HIF1α is responsible for glycolysis and OXPHOS in T_FH_ cell differentiation and GC responses. Supplementary Figure S3: Alterations of glycolysis and OXPHOS signaling controls T_FH_ cell differentiation upon PR8 virus infection. Supplementary Figure S4: Alterations of glycolysis and OXPHOS signaling controls T_FH_ cell differentiation upon PR8 virus infection. Supplementary Figure S5: Regulations of glycolytic activities on homeostasis and differentiation of T_FH_ cells in anti-virus immunity.
