# Supplementary material for: HIF1α-Dependent Metabolic Signals Control the Differentiation of Follicular Helper T Cells
Source: Cells. 2019 Nov 17;8(11):1450. doi: 10.3390/cells8111450 (PMC6912655; doi:10.3390/cells8111450)
Supplement: Supplementary file 1 [file cells-08-01450-s001.pdf]

Supplemental Figure

HIF1α-dependent metabolic signals control the differentiation of follicular helper T cells

Lin Dong[1,#], Ying He[1,#], Shuping Zhou[2,#], Yejin Cao[1], Yan Li[1], Yujing Bi[3,*], Guangwei Liu[1,*]

[1]Key Laboratory of Cell Proliferation and Regulation Biology, Ministry of Education, Institute of Cell Biology, College of Life Sciences, Beijing Normal University, Beijing China; [2]Institute of Basic Medicine, Shandong First Medical University & Shandong Academy of Medical School, Jinan China; [3]State Key Laboratory of Pathogen and Biosecurity, Beijing Institute of Microbiology and Epidemiology, Beijing China

Fig. S1

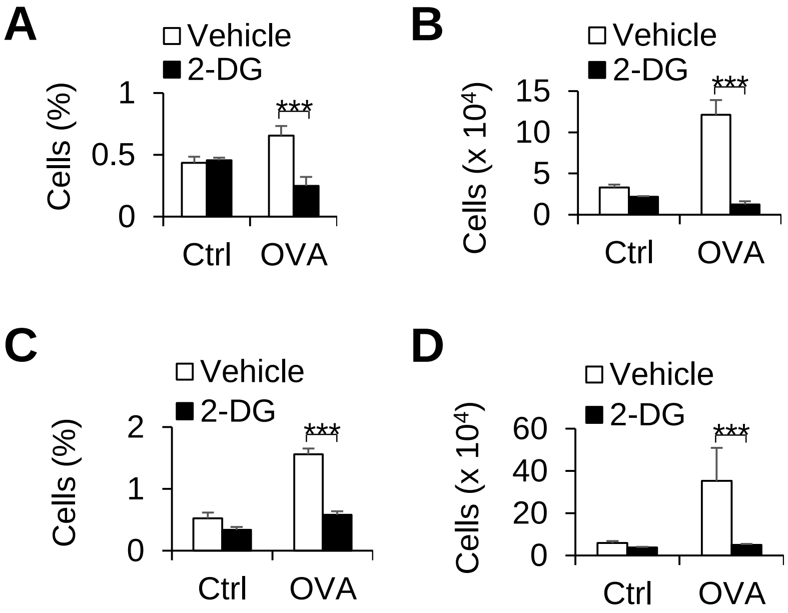

Figure S1. Blocking glycolysis inhibits T_{FH} cell differentiation upon foreign antigen stimuli.
Flow cytometry analysis of plasma cells and IL-21^+ CXCR5^+T_{FH} cells in spleen. The percent and absolute number of plasma cells (**A** and **B**) and IL-21^+CXCR5^+T_{FH} cells (**C-D**) are shown. ***P< 0.001, compared with the indicated groups.

Fig. S2

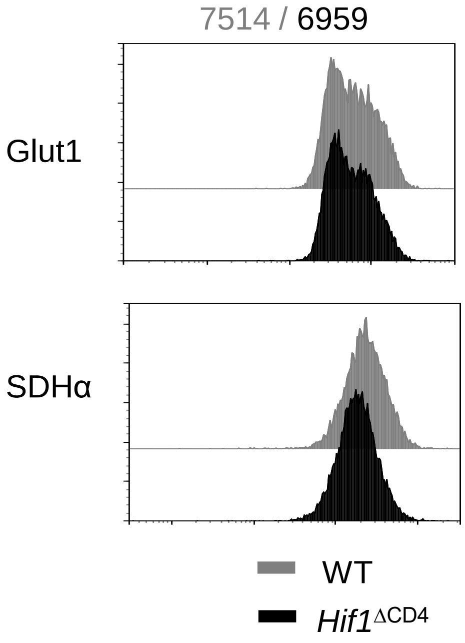

Figure S2. HIF1α is responsible for glycolysis and OXPHOS in T_{FH} cell differentiation and GC responses.
Flow cytometry analysis of Glut1 and SDHα expression of T_{FH} cells in spleen from WT and HIF1α-deficient mice at 8 days after OVA-immunization.

Fig. S3

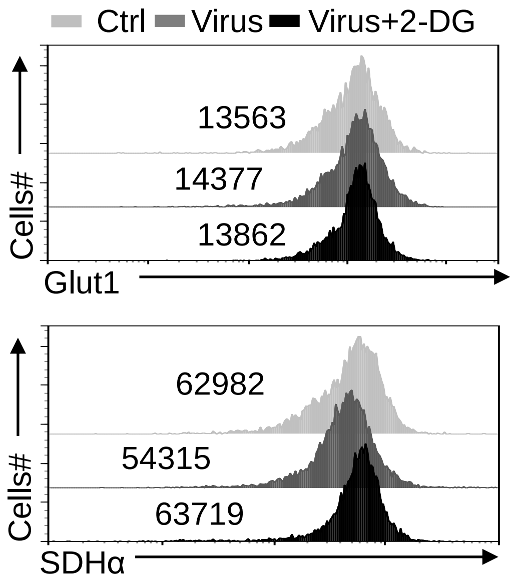

Figure S3. Alterations of glycolysis and OXPHOS signaling controls T_{FH} cell differentiation upon PR8 virus infection. Flow cytometry analysis of Glut1 and SDHα expression of T_{FH} cells in lung from PR8-infected mice at 8 days in the presence of 2-DG treatment. .

Fig. S4

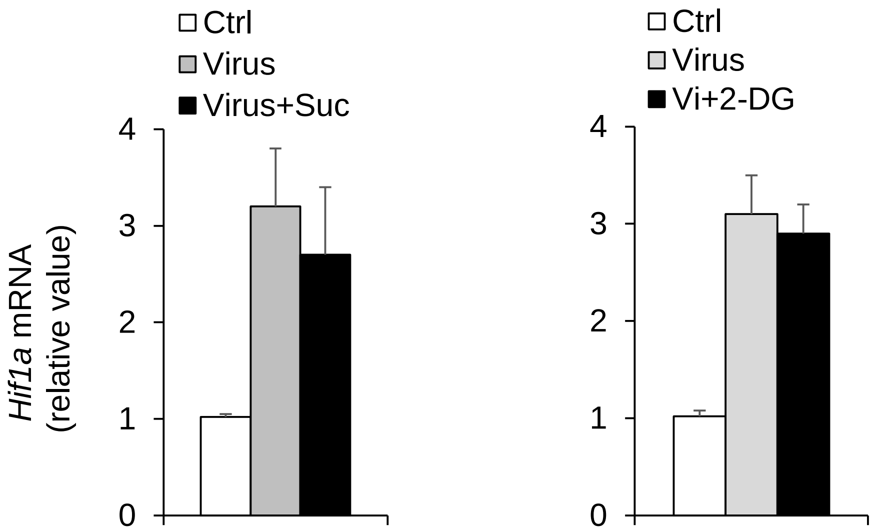

Fig. S4. Alterations of glycolysis and OXPHOS signaling controls T_{FH} cell differentiation upon PR8 virus infection. Real-time PCR of *Hif1a* mRNA in T_{FH} cells sorted from lung from PR8-infected mice at 8 days in the presence of 2-DG treatment.

Fig. S5

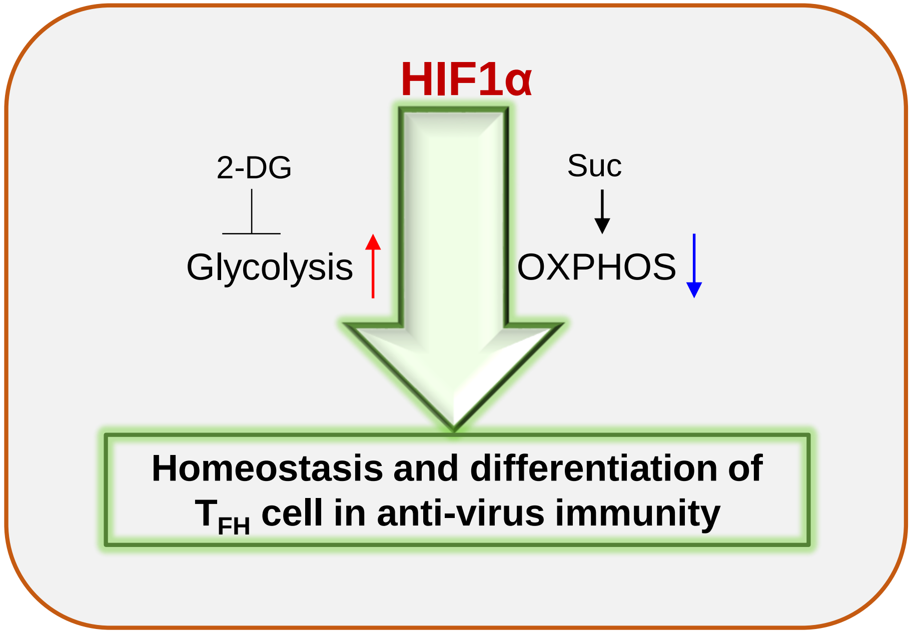

Fig. S5 Regulations of glycolytic activities on homeostasis and differentiation of T_{FH} cells in anti-virus immunity.
Proposed model how glycolysis and OXPHOS in T_{FH} cells integrate the adaptive stimuli to regulate the GC responses and T_{FH} cell differentiation under steady state or antigen immunization even in anti-virus immunity through metabolic dependent mechanisms.
